# Psychosocial working conditions, perceived patient safety and their association in emergency medical services workers in Germany – a cross-sectional study

**DOI:** 10.1186/s12873-024-00983-2

**Published:** 2024-04-14

**Authors:** Antonia Elsässer, Annegret Dreher, Reinhard Pietrowsky, Frank Flake, Adrian Loerbroks

**Affiliations:** 1 Institute of Occupational, Social and Environmental Medicine, Centre for Health and Society, Faculty of Medicine, University of Düsseldorf, Moorenstr. 5, 40225 Düsseldorf, Germany; 2 Department of Experimental Psychology, Heinrich Heine University Düsseldorf, Düsseldorf, Germany; 3 German Association of Emergency Medical Services (Deutscher Berufsverband Rettungsdienst e. V.), Lübeck, Germany

**Keywords:** Emergency medical service, Germany, Health care workers, Occupational stress, Psychosocial working conditions, Quality of care

## Abstract

**Background:**

Emergency medical service (EMS) workers face challenging working conditions that are characterized by high stress and a susceptibility to making errors. The objectives of the present study were (a) to characterize the psychosocial working conditions of EMS workers, (b) to describe the perceived quality of patient care they provide and patient safety, and (c) to investigate for the first time among EMS workers associations of psychosocial working conditions with the quality of patient care and patient safety.

**Methods:**

For this cross-sectional study, we carried out an online survey among 393 EMS workers who were members of a professional organization. Working conditions were measured by the Demand-Control-SupportQuestionnaire (DCSQ) and seven self-devised items covering key stressors. Participants reported how often they perceived work stress to affect the patient care they provided and we inquired to what extent they are concerned to have made a major medical error in the last three months. Additionally, we used parts of the Emergency Medical Services – Safety Inventory (EMS-SI) to assess various specific errors and adverse events. We ran descriptive analyses (objective a and b) and multivariable logistic regression (objective c).

**Results:**

The most common stressors identified were communication problems (reported by 76.3%), legal insecurity (69.5%), and switching of colleagues (48.9%) or workplaces (44.5%). Overall, 74.0% reported at least one negative safety outcome based on the EMS-SI. Concerns to have made an important error and the perception that patient care is impaired by work stress and were also frequent (17.8% and 12.7%, respectively). Most psychosocial working conditions were associated with the perception that patient care is impaired due to work stress.

**Conclusions:**

Work stress in EMS staff is pronounced and negative safety outcomes or potential errors are perceived to occur frequently. Poor psychosocial working conditions were only consistently associated with perceived impairment of patient care due to work stress. It seems necessary to reduce communication problems and to optimize working processes especially at interfaces between emergency services and other institutions. Legal insecurity could be reduced by clarifying and defining responsibilities. Communication and familiarity between team colleagues could be fostered by more consistent composition of squads.

**Supplementary Information:**

The online version contains supplementary material available at 10.1186/s12873-024-00983-2.

## Background

Emergency medical services (EMS) workers perform crucial health care tasks in terms of fast and accurate life-saving treatment of acute and critically ill patients as well as attentive monitoring of patients during transport to the hospital [1]. However, just as other health professionals [2–4], EMS workers face challenging psychosocial working conditions that are characterized by high stress and a susceptibility to making errors [5–7]. Such errors can have serious consequences for patients’ health and prognosis [8].

Important stressors in EMS are, for example, the challenge to work under considerable time pressure and a high responsibility in terms of diagnostic and treatment choices [9, 10]. At the same time though EMS staff lacks the diagnostic and therapeutic assistance and psychosocial support that medical workers in the clinical setting can often rely on [11]. The work of EMS workers is further characterized by little control, for instance, related to their work schedules and decision-making in the EMS station [12]. The above-mentioned mix of challenges and resulting work stress can be best conceptualized in terms of the well-established Demand-Control-Support (DCS) model. The DCS model defines work stress as the combination of high psychological demands with low decision latitude (i.e., the control over one’s tasks and when and how to complete them) as well as low social support at work [13, 14].

However, EMS workers’ daily work is also affected by job-specific stressors which are *no*t captured by the DCS model or other dominant work stress models (e.g., the model of organizational justice [15] or the effort-reward imbalance model [16]). Such job-specific stressors include, for instance, working procedures and protocols that may vary considerably between emergency departments and that can differ several times in a single shift [17]. Also, EMS workers often seem to face communication problems, in particular with colleagues or staff at the control center or hospitals [1, 18]. Furthermore, working in unsafe environments and public spaces with the possibility of third parties watching or interfering and even attacking them is reported as stressful for EMS workers [9, 19]. In addition, the legal framework for the scope of EMS’ tasks and responsibilities - at least in Germany - is perceived as being vague and EMS workers feel uncertain in terms of what they are legally allowed or required to do in an emergency situation [9, 19]. Further relevant stressors may include shift work, being on call constantly and traumatic experiences in action [18, 20].

Research has repeatedly shown that work stress increases the risk of poor physical and mental health [21]. Accordingly, studies investigating the health status of EMS workers report high prevalences of insomnia, posttraumatic stress disorder, depression as well as anxiety, which occur more frequently than in the general population [17, 22, 23]. Also, among the population of health professions, EMS workers seem to display particularly poor mental health, e.g., in terms of burnout scores [24].

Across various health professions, poor mental health has been associated with poorer patient safety [25–27]. The same seems to hold true for EMS workers, as studies suggest that burnout [28] and sleeping disorders [11] among EMS are associated with poorer patient safety. These previous studies among EMS workers have primarily addressed psychophysiological reactions to stressful working conditions (e.g., burnout) as determinants for the quality of patient care and patient safety [11, 28]. However, evidence on the possible association of specifically working conditions (that may contribute to subsequent burnout) with patient safety in EMS workers is lacking. Such an association has already been investigated and confirmed for other health professions, such as physicians, nurses and medical assistants [29–32].

The objectives of the present study were (a) to characterize the psychosocial working conditions of EMS workers in terms of both the DCS model and EMS-specific stressors, (b) to describe the perceived quality of patient care they provide and patient safety, and (c) to investigate for the first time associations of those psychosocial working conditions with the perceived quality of patient care and patient safety. Research into the potential links between psychosocial working conditions and the quality of patient care among EMS workers may inform the development of early interventions that improve working conditions and thereby increase the likelihood of optimal care being delivered.

## Methods

### Study setting and population

We carried out a cross-sectional study among EMS workers who were recruited through the social media channels of the German Association of Emergency Medical Service (Deutscher Berufsverband Rettungsdienst e.V. [DBRD]). In Germany, over 85,000 individuals are employed as EMS workers [33] and approximately 10,000 of those are members of the DBRD [34]. These members were invited to participate in an online survey on the platform “Unipark” on 15th of September 2021. A reminder to participate was sent on 19th of November 2021 and the survey was closed on the 27th of November 2021. The inclusion criteria for participants (applied after data collection) were 1) an age of 18 years or older and 2) current employment in the EMS field.

### Questionnaire

We developed our questionnaire in collaboration with representatives of DBRD, who are experienced EMS workers themselves and are in close contact with a large number of EMS workers.

### Working conditions

Working conditions were measured with the Demand-Control-Support Questionnaire (DCSQ) [13, 14], which consists of 17 items and three subscales. Those scales measure psychological demands (five items), decision latitude (six items), and social support at work (six items) [35]. All 17 items are expressed as statements with a four-point Likert scale inquiring after the level of agreement. All subscale scores are calculated by summing up the respective item scores while reversing the scoring of item 4 (overtime work) and item 9 (variety of work) [14]. Higher values indicate higher psychological demands (potential score range 5–20), higher decision latitude (potential score range 6–24), and higher social support at work (potential score range 6–24). For our study, we used the German-language version of the DCSQ by Mauss et al. [14]. Since some important stressors identified in the literature are not covered by the DCSQ (see introduction), we developed seven additional items covering communication, changing working environments, the experience of working in public spaces, legal insecurity and changing team partners as potential stressors (for the wording of items, response format and sources see additional file 1, Table A). We reviewed and revised the questions in cooperation with the DBRD to maximize the likelihood that they are easily comprehensible and that they cover relevant everyday challenges of EMS workers.

### Patient safety

To measure patient safety, we used the following items: (a) “Are you concerned that you have made a major medical error in the last three months?” (yes, no) [36], and (b) “How often do you think work stress affects your work with and on patients?” (never, rarely, occasionally, mostly, always) [37].

Additionally, we built on parts of the 44-item Emergency Medical Services Safety Inventory (EMS-SI), which was developed by Patterson et al. [11] specifically for the EMS sector. We used the 25 items measuring medical errors and adverse events which are available in German [28]. These include typical tasks and possible related errors made by EMS workers in prehospital treatment and during transport of the patient, e.g., when establishing an intravenous (IV) or intraosseous (IO) access, during airway intervention, monitoring, when choosing medication, during provision of first aid wound care, when making diagnoses and treatment decisions, or when transporting and admitting the patient to the right hospital. All items refer to events that occurred in the three months prior to filling out the questionnaire.

The items are subdivided into two sections using different response formats, both measuring the occurrence of adverse events:

- Section 1 (17 items) inquires after different behaviors in specific situations that might pose an adverse event (e.g. “I did not check a glucose level in a patient with altered mental status because…”). Respondents are then to specify the reason for their behavior in that situation. The response options are “ran out of time,” “forgot to perform,” “not part of protocol,” “did not think it necessary,” “contraindicated,” “do not wish to answer,” and “not applicable to me.”
- Section 2 (8 items) covers possible adverse events, but does not inquire after the reasons, but after the occurrence of those events (e.g. “…I made a patient with chest pain ambulate instead of using a stretcher.”). The response options are “definitely not,” “probably not,” “I’m not sure,” “probably yes,” “definitely yes,” “do not wish to answer,” or “not applicable to me“.

After discussion of the questionnaire with the DBRD, we added an additional response option to the items of section 1 that was considered important for German EMS workers, but was missing in the original version [11] as well as the German version of the EMS-SI [28]. This additional response option was “the measure was not authorized by the physician in charge”. The following responses were considered indicators of negative patient safety outcomes (NSOs) caused by the responding EMS worker:

- Section 1: “ran out of time”, “forgot to perform”, and “did not think it was necessary”.
- Section 2: “probably yes” and “definitely yes”.

### Additional information

To describe the study population and to be able to adjust for relevant confounders, the questionnaire additionally covered, amongst others, the following aspects:

- Demographics: sex, age, and highest level of school education.
- Health status: overall self-rated current health measured by a 5-point Likert scale with responses options ranging from “very good” to “poor”.
- Occupational factors:

- highest level of paramedic training (EMT-Paramedic/”Notfallsanitäter” = 3 year training; EMT-Paramedic/“Rettungsassistent“ = 2 year training; EMT-Intermediate“Rettungssanitäter“ = 520 h training; EMT-Basic/“Rettungshelfer” = 320 h training; none of the above),
- years worked in the paramedic sector, and
- current employer (response options included the largest federal and private employers for paramedics in Germany as well as the German Armed Forces).

### Statistical analyses

We ran descriptive analyses for all variables displaying absolute numbers and percentages. For the DCSQ, the scores for each subscale (i.e., psychological demands, decision latitude and social support at work) were added after reverse scoring of item 19 (overtime work) and item 25 (variety of work). A median split was used to operationalize low and high levels of each subscale [14]. Work stress according to the DCSQ was defined as the simultaneous occurrence of high psychological demands, low decision latitude and low social support at work. Our newly developed items related to job-specific stressors were each dichotomized into “agree” and “disagree”.

The original 5-point answer scale of the item “How often do you think work stress affects your work with and on patients?” was dichotomized to capture impairment of patient care, that is, “always”/”often” versus “occasionally”/ “rarely” /“never”. The EMS-SI was analyzed regarding the occurrence of negative safety outcomes (NSOs). Therefore, all answers that were classified as indicators for a NSO were summed across scales to determine the number of NSOs for each participant. For logistic regression, we used a median split to categorize them into few/none (≤ 2) and many (> 2) NSOs. Additionally, the different responses reflecting a NSO were counted to identify the most common answers or reasons for possible errors.

We ran logistic regression using the DCSQ as well as job-specific stressors as the exposures. We ran three separate logistic regressions, that is, for each of the following outcomes:

1. concerns to have made an important error in the in the last three months (yes vs. no),
2. impairment of patient care (yes vs. no), and
3. NSO according to the EMS-SI categorized into many (> 2) vs. few/none (≤ 2).

All models were adjusted for age, gender and level of paramedic training (high = 2- or 3-year training vs. low = < 2-year training). Although years in the paramedic field was also identified as a possible confounder, we did not adjust for it as it was highly correlated with age (Pearson correlation coefficient > 0.8). Statistical analyses were conducted using IBM SPSS Statistics 29.0.1.0.

To explore the robustness of our findings, we also dichotomized the DCSQ subscales based on the top or the bottom tertile of the distribution in sensitivity analyses. These multivariable analyses suggested similar patterns of associations as logistic regression analyses with variables dichotomized according to the median split (data not shown)

## Results

### Descriptive analysis

#### Demographics and participant characteristics

A total of 401 EMS workers participated in this study. After removal of one participant with missing data and seven who stated they were not actively working in the paramedic field at the time of data collection, the remaining 393 participants were included in the study. Characteristics of the study population are displayed in Table 1. As much as 83.0% of the participants were male and the median participant age was 32 years (interquartile range 26–41). Over half of the participants reported a high educational level and 82.7% of the respondents completed either a paramedic training of “Nofallsanitäter*in” (3-year training) or “Rettungsassistent*in” (2-year training). About half of the respondents rated their health as “very good” or “good”.

**Table 1:** Characteristics of the study sample

Characteristics	Total (*n* = 393)
Sex, n (%)	
Male	326 (83.0)
Female	65 (16.5)
Diverse/not specified	2 (0.5)
Age in years, median (IQR^1^)	32 (26-41)
Age in years, n (%)	
18–29	157 (39.9)
30–39	128 (32.6)
40–49	81 (20.6)
50–59	23 (5.9)
60 and older	4 (1.0)
Highest level of education, n (%)	
Low ^2^	13 (3.3)
Intermediate ^3^	155 (39.4)
High ^4^	221 (56.2)
Other	4 (1.0)
Highest level of paramedic training, n (%)	
3 year training (“Notfallsanitäter*in”)	315 (80.2)
2 year training (“Rettungsassistent*in“)	10 (2.5)
520 h training (“Rettungssanitäter*in“)	64 (16.3)
320 h training („Rettungshelfer*in“)	3 (0.8)
None of the above	1 (0.3)
Years worked in the paramedic sector, median (IQR^1^)	10 (5-16)
Years worked in the paramedic sector, n (%)	
1–5	101 (25.7)
6–10	118 (30.0)
11–15	69 (17.6)
16–20	37 (9.4)
21–40	68 (17.3)
Current employer, n (%)	
Aid organizations^5^	281 (71.5)
Fire Brigade	49 (12.5)
Military	1 (0.3)
Other	62 (15.8)
Type of employment, n (%)	
Full-time	344 (87.5)
Part-time	33 (8.4)
Marginal employment	7 (1.8)
Volunteer	6 (1.5)
Other	3 (0.8)
Self-rated health, n (%)	
Very good	39 (9.9)
Good	164 (41.7)
Satisfactory	134 (34.1)
Not so good	52 (13.2)
Poor	4 (1.0)

^1^IQR = Interquartile range; ^2^Secondary modern school qualification (‘Haupt-/Volksschulabschluss‘); ^3^Secondary school level 1 certificate (‘Mittlere Reife‘, ‘Realschulabschluss‘ or ‘Fachschulreife‘); ^4^General qualification for university entrance (‘Abitur‘) or entrance qualification limited to universities of applied sciences (‘Fachhochschulreife‘); ^5^ Workers Samaritan Federation, German/Bavarian Red Cross, Johanniter Unfall-Hilfe, Malteser Hilfsdienst

#### DCSQ and job-specific stressors

As shown in Table 2, high work stress according to the DCS model was reported by 13.7% of the participants. Considering the respective potential score ranges, demands seemed to be at intermediate levels (i.e., a mean score of 13.6 given a potential score range of 5–20), but decision latitude and social support were comparatively somewhat more pronounced.

**Table 2:** Work stress according to the demand-control-support questionnaire and job-specific stressors of EMS workers

Variable	Total (*n* = 393)
DCSQ^1^	
Psychological demands^2^, median (IQR^3^), mean	14 (12-15), 13.6
High, n (%)	204 (51.9)
Low, n (%)	189 (48.1)
Decision latitude^2^, median (IQR^3^), mean	17 (16-19), 17.0
High, n (%)	239 (60.8)
Low, n (%)	154 (39.2)
Social support at work^2^, median (IQR^3^), mean	17 (15-19), 17.2
High, n (%)	239 (60.8)
Low, n (%)	154 (39.2)
Work stress^4^	
High, n (%)	54 (13.7)
Low, n (%)	339 (86.3)
Perceived stressors^5^, n (%)	
Communication…	300 (76.3)
with clinics or nursing homes	352 (89.6)
at the EMS^6^station	141 (35.9)
with the control center	297 (75.6)
with the police or fire department	89 (22.6)
with patients	236 (60.1)
with patients’ relatives	282 (71.8)
Switching of colleagues	192 (48.9)
Switching of workplaces	175 (44.5)
Working in a public space	86 (21.9)
Legal insecurity	273 (69.5)

^1^DCSQ = Demand control support questionnaire; ^2^Categorized into high and low at the respective median split; ^3^IQR = Interquartile range; ^4^High work stress = simultaneous occurrence of high psychological demands, low decision latitude and low social support at work; ^5^ job-specific stressors were each dichotomized into “agree” and “disagree”; ^6^EMS = Emergency Medical Service

Regarding job-specific stressors, communication was most often perceived as a stressor, especially with staff at clinics or nursing homes, the control center, patients and their relatives. Legal insecurity was reported as a stressor by 69.5%.

#### Patient safety

As shown in Table 3 as much as 12.7% of the participants reported subjective impairment of patient care due to work stress and 17.8% were concerned they had made an important error in the past three months. According to the EMS-SI 74.0% reported at least one negative safety outcome during that time.

**Table 3:** Description of patient safety indicators

Questionnaire items	Total (n = 393)
How often does work stress affect your work with and on patients?, n (%)	
Never	6 (1.5)
Rarely	109 (27.7)
Occasionally	228 (58.0)
Often	45 (11.5)
Always	5 (1.3)
Impairment of patient care^1^	50 (12.7)
Are you concerned you made an important error in patient care in the last three months?, n (%)	
Yes	70 (17.8)
No	323 (82.2)
EMS-SI^2^	
Median number of negative safety outcomes^3^ (IQR^4^)	2 (0–3)
Occurrence of at least one negative safety outcome^3^, n (%)	219 (74.0)
Occurrence of individual negative safety outcomes^3^, each as n (%):	
Did not print and properly interpret a 6-inch EKG^5^ strip	136 (34.6)
Made a patient with chest pain ambulate instead of using a stretcher	128 (32.6)
Did not administer the necessary treatment for a specific condition/malady	112 (28.5)
Did not establish an IV^6^ after two attempts	60 (15.3)
Did not use a secondary treatment device when the preferred failed	51 (13.0)
Did not check a glucose level in a patient with altered mental status	44 (11.2)
Did not place a patient on the monitor	42 (10.7)
Did not transport a specialty care patient to a specialty care facility	35 (8.9)
Did not perform a 12-lead EKG^5^ on a patient with chest pain	32 (8.1)
Did not check a glucose level in a diabetic patient with nausea and vomiting	23 (5.9)
Accidentally caused physical injury to a patient moving the patient	21 (5.3)
Administered the wrong dose of medication by not confirming the dose	17 (4.3)
Did not properly size a piece of equipment and then used it on a patient	16 (4.1)
Did not intubate a patient in respiratory arrest	15 (3.8)
Accidentally started an IO^7^ in a location outside of protocol	14 (3.6)
Administered the wrong medication by not checking the label	11 (2.8)
Did not perform an airway intervention on a patient with congestive heart failure while enroute to the hospital	10 (2.5)
Accidentally dropped a patient while on a transportation device	10 (2.5)
Accessed a dialysis port or other vascular device outside of protocol	8 (2.0)
Placed an IV^6^ into an artery instead of into a vein	7 (1.8)
Confirmed a STEMI^8^ but did not administer aspirin when warranted	5 (1.3)
Did not perform a 12-Lead EKG^3^ on a patient with STEMI^8^	2 (0.5)
Did not secure an embedded object in a wound instead of securing the object with bandages and accidently removed it	3 (0.8)
Transferred a patient at the emergency department with an unrecognized esophageal intubation	1 (0.3)
Accidentally dislodged an ET^9^ tube	1 (0.3)

^1^ The answers “often” and “always” are considered an impairment of patient care; ^2^EMS-SI = Emergency medical services safety inventory; ^3^ The answers “Probably Yes,” “Definitely Yes,” “Ran Out of Time,” “Forgot to Perform,” and “Did Not Think it was Necessary” are indicators for negative safety outcomes; ^4^ IQR = Interquartile range; ^5^ EKG = Electrocardiogram; ^6^ IV = intravenous access; ^7^ IO = intraosseous access; ^8^ STEMI = ST-elevation myocardial infarction; ^9^ ET = Endotracheal

The most common NSOs (reported by at least 28% of the sample) were not printing and properly interpreting a 6-inch EKG strip, making a patient with chest pain ambulate instead of using a stretcher as well as not administering the necessary treatment for a specific condition or complaint. The most frequently reported reason for a negative safety outcome was thinking that the task in question was not necessary (33.3%) (see Table 4).

**Table 4:** Most frequently reported answers representing negative safety outcomes vs. no negative safety outcomes accoring to the EMS-SI^1^

Answers given	Total (*n* = 866)
NSO^2^, n (%)	503 (58.1)
did not think it was necessary	288 (33.3)
ran out of time	120 (13.9)
forgot about it	95 (11.0)
No NSO^2^, n (%)	363 (41.9)
the measure was not authorized by the physician in charge	191 (22.1)
not part of protocol	86 (10.0)
contraindicated	47 (5.4)
do not wish to answer	39 (4.5)

^1^EMS-SI = Emergency medical services safety inventory; ^2^NSO = Negative safety outcomes

The answer “not applicable to me” with *n* = 5707 of 6573 (86.8%) is not shown in this table

### Associations of psychosocial working conditions and patient safety

The results from all threemultivariable logistic regression analyses are shown in Table 5.

**Table 5:** Associations between psychosocial working conditions and patient safety indicators (multivariable logistic regression)

	Impairment of patient care^3^ OR^6^ (95% CI^7^)	Concerns to have made an important error^4^ OR^6^ (95% CI^7^)	EMS-SI Few vs. many NSOs^2, 5^ OR^6^ (95% CI^7^)
DCSQ^1^			
Psychological demands^8^			
High (vs. low)	**10.16 (3.93–26.26)**	**1.73 (1.01–2.96)**	1.48 (0.96–2.30)
Decision latitude^8^			
High (vs. low)	**0.45 (0.24–0.82)**	0.60 (0.35–1.01)	0.70 (0.45–1.09)
Social support at work^8^			
High (vs. low)	**0.24 (0.12–0.45)**	0.78 (0.46–1.34)	0.71 (0.45–1.11)
Work stress^9^ High (vs. low)	**5.48 (2.73–10.87)**	1.49 (0.71–3.14)	1.02 (0.54–1.95)
Perceived stressors			
Switching of colleagues			
Yes (vs. no)	1.11 (0.58–2.10)	**1.81 (1.01–3.23)**	1.30 (0.81–2.07)
Changing workplace			
Yes (vs. no)	**4.04 (2.08–7.86)**	1.53 (0.90–2.59)	**1.61 (1.04–2.49)**
Working in a public place			
Yes (vs. no)	**1.96 (1.03–3.75)**	**1.90 (1.04–3.45)**	1.32 (0.79–2.21)
Legal insecurities			
Yes (vs. no)	**4.00 (1.64–9.75)**	1.66 (0.88–3.10)	1.28 (0.79–2.06)
Communication			
Yes (vs. no)	**3.98 (1.39–11.41)**	1.37 (0.72–2.63)	**1.96 (1.12–3.43)**
with clinics or nursing homes			
Yes (vs. no)	1.80 (0.53–6.11)	1.83 (0.62–5.40)	**2.59 (1.05–6.43)**
at the EMS^10^-station			
Yes (vs. no)	1.66 (0.90–3.06)	1.51 (0.88–2.62)	0.92 (0.58–1.46)
with the control center			
Yes (vs. no)	**3.19 (1.21–8.37)**	1.11 (0.60–2.04)	1.19 (0.71–2.01)
with the police or fire department			
Yes (vs. no)	1.42 (0.72–2.79)	0.84 (0.44–1.61)	0.79 (0.46–1.36)
with patients			
Yes (vs. no)	**2.53 (1.25–5.12)**	1.15 (0.67–1.70)	1.23 (0.78–1.93)
with patients’ relatives			
Yes (vs. no)	**3.88 (1.50-10.09)**	1.60 (0.86-3.00)	**1.86 (1.10–3.32)**

Statistically significant findings (*p* < 0.05) are highlighted in bold letters

All models were adjusted for age, gender and level of paramedic training (high = 2- or 3-year training vs. low = < 2-year training)

Psychosocial working conditions: measured with DSCQ and job-specific stressors

Patient safety: measured with subjective impairment of patient care, concerns to have made an important error and EMS-SI

^1^DCSQ = Demand-Control-Support Questionnaire; ^2^EMS-SI = Emergency medical services safety inventory; ^3^ The answers “often” and “always” are considered an impairment of patient care; ^4^ Occurrence within the past three months; ^5^ NSO = Negative Safety Outcomes. The answers “Probably Yes,” “Definitely Yes,” “Ran Out of Time,” “Forgot to Perform,” and “Did Not Think it was Necessary” are indicators for negative safety outcomes; ^6^Odds ratio; ^7^Confidence interval; ^8^ Categorized into high and low through median split; ^9^ High work stress = simultaneous occurrence of high psychological demands, low decision latitude and low social support at work; ^10^Emergency Medical Service

#### Impairment of patient care due to work stress

Overall, the vast majority of psychosocial working conditions were associated with the perception that patient care is impaired due to stress. In particular the DCS subscales showed strong associations with that outcome. For instance, work stress in terms of the DCS model was associated with a more than 5-fold increased odds of reporting impaired patient care due to work stress (95% CI = 2.7–10.9). A pattern of positive associations was also found for job-specific stressors: ORs of 1.7 and above were found for 9 out of 11 stressors. Reported impairment of patient care was particularly more common in participants who reported changing workplaces, legal insecurities, and communication problems with the control center or patients’ relatives (OR≥ 3.2).

#### Concerns of having made an important error

Altogether, many psychosocial working conditions showed a moderate association with the concern to have made an important error in the past 3 months. However, only the positive associations with high psychological demands, switching of colleagues and working in public places reached statistical significance.

#### Negative safety outcomes

Psychosocial working conditions were mostly weakly and inconsistently associated with negative safety outcomes. The ones that showed statistical significance were the following: participants who stated that they perceive frequent changing of the workplace as a stressor were more likely to report more than two negative safety outcomes in the EMS-SI (OR 1.6 [CI 1.04–2.5]). Also communication, especially with clinics or nursing homes as well as patients’ relatives were significantly associated with a higher number of negative safety outcomes (see Table 5).

## Discussion

### Psychosocial working conditions and work-related stressors

We examined working conditions among EMS workers in terms of both, the established DCS model and job-specific factors. We generally found high levels of agreement to the latter stressors which illustrates that we identified relevant topics for EMS workers in Germany that are not covered by the dominant work stress instruments. Among the newly developed items especially communication problems and legal insecurity were reported as common stressors.

Communication issues were perceived as a stressor by over 75% of our participants, particularly with staff at clinics or nursing homes, the control center, patients or their relatives. Communication issues with the EMS station or the police and fire department were reported less frequently. With regard to the police and fire departments, fewer communication problems may simply be due to less frequent interaction in everyday EMS work as compared to clinics, control centers and patients.

Legal insecurity was reported as a stressor by 69.5% of the participants. Despite the highest level of EMS training, “Notfallsanitäter*innen” do not have the same legitimation to administer medication and execute procedures as a physician. In emergency situations EMS workers may face a dilemma and they often have to choose between on the one hand violation of authority structures and legal frameworks and on the other hand the failure to provide required emergency care [38]. While they might want to and even would be able to help, they are not sure if they are allowed to, which can be described with the concept of moral distress [39].

Work stress according to the DCSQ was only reported by 13.7% of the participants. This is in keeping with a study by Alexander et al. [40] who reported high job satisfaction in ambulance personnel. In that 2001 study, a distinction was made between satisfaction with the job itself and satisfaction with how the system operates, so called organizational satisfaction, with the latter being generally low in EMS workers [41]. Other studies that used different measures to capture work stress, however, report high work stress in paramedics [7, 10, 42, 43]. Bardhan et al. [42] and Grochowska et al. [10] used parts of the Effort-Reward-Imbalance model to determine work stress and reported a high effort-reward ratio and overcommitment as well as excessive duties and high responsibility. Van der Ploeg et al. [7] used the subscale “high emotional demands” of the “Questionnaire on the Experience and Assessment of Work” and reported that the EMS workers perceived their job as more emotional demanding than staff in various other health services used as a reference group.

In our study, we additionally included decision latitude as well as social support. Social support is believed to potentially serve as a buffer against stress at work [13, 44] and could explain the generally low prevalence of work stress found in our study. Another study also using the DCSQ amongst EMS workers [45] suggested similar findings to ours, with the score for psychosocial factors being close to the mid-point of the potential score range for psychological demands and higher for decision latitude and social support in both studies, therefore suggesting a generally positive psychosocial work environment in the ambulance service.

### Patient safety

Impairment of patient care due to work stress and concerns to having made an important error in the past three months were reported by 12.7% and 17.8% of participants, respectively. A study among nurses [46] used a longer reference period (i.e., the last year). Accordingly, that study yielded higher numbers: the majority of nurses (72.7%) stated that they had made mistakes without negative consequences for patients, while 33.5% reported that they had made mistakes with negative consequences to patients. With regard to the impairment of patient care that prior study captured different dimensions, using the questions “I fall short in the quality of care I provide to my patients” and “I do not have enough time or attention for my patients”, with 26.2% respectively 51% of the nurses agreeing to those statements. In our study sample those questions were not posed, but could have explained the nature of the impairment of patient care more precisely. In posing similar questions, it would have been possible to differentiate different dimensions of patient care, e.g. if only time with patients was shortened or if the quality of the interaction between patient and EMS worker was impaired. Those aspects could be covered in future studies investigating the impairment of patient care in EMS workers.

High numbers were found in a study amongst paramedics in Germany concerning patient safety [1]: when asking if participants had harmed a patient through their work in the course of their career, 72.0% of the participants affirmed to this statement. Again, these high numbers compared to our study could stem from the fact that Zimmer et al. inquired after the course of one’s whole career compared to only the three months in our study.

Based on the EMS-SI, 74.0% of our study participants reported at least one negative safety outcome. These findings are in close keeping with those of Baier et al. [28] who used the same tool in EMS workers in Germany who were recruited through various social media channels, journals as well as through the DBRD. Among their 1101 participants, 73.7% reported at least one negative safety outcome. Additionally, the four most common NSOs were in line with the ones in our study which increases confidence in the validity of our findings.

The noteworthy difference between participants reporting a subjective impairment of patient care due to work stress as well as being concerned to have made an important error (12.7% and 17.8%, respectively) and the results of the EMS-SI (at least one NSO in 74.0%) needs to be discussed. The fact that the most frequently chosen response category to justify errors identified through the EMS-SI was “did not think it was necessary” could explain the putative mismatch between the different approaches to measuring patient safety: if the participants do not perceive a specific measure or task as necessary, it is easy to conceive that they would not consider the failure to carry out that task an important error. As the item inquiring after the concern of having made an important error was presented before specific errors using the EMS-SI in the questionnaire, it can be assumed participants were not influenced in their response behavior by the sequence of questions.

It has been reported that the errors and adverse events measured by the EMS-SI were originally assembled and developed by EMS medical directors, emergency medical technicians and paramedics as well as epidemiologists [11]. The EMS-SI was thoroughly discussed with members of the DBRD in order to adapt it to EMS workers in Germany for both this study and the study of Baier et al. [28]. Thus, it seems unlikely that that tool’s validity is dramatically poor. It rather seems that EMS workers have an incomplete understanding of the range and nature of errors associated with their day-to-day occupational task and, if so, this observation implies further training need.

### Associations of psychosocial working conditions and patient safety

To our knowledge, the present study is the first to investigate the possible association between psychosocial working conditions and patient safety among EMS workers. Psychosocial working conditions showed a pattern of consistent and fairly pronounced associations with perceived impairment of patient care due to stress. This held true for both the established DCS model as well as the novel items we devised. Findings were less consistent with regard to the analyses pertaining to concerns to have made an important medical error and with regard to the NSOs. A contributing factor may be that our sample was too small to detect modest associations with statistical significance. Also, we cannot rule out that several significant associations are due to chance. However, we will discuss the key associations that we found.

The experience of frequent switching of colleagues was associated with concerns of having made an important error in the last three months. When not working together often, team mates are likely less familiar with each other`s workflow and can make for a less coordinated team. Accordingly, the evaluation of the German Critical Incident Reporting System for Emergency Medicine identified a deficit in team communication as the trigger for 27% of cases of patient harm [47].

Participants who perceived working in a public place as a stressor were also more likely to report concerns of having made an important error while this was not the case for our other outcomes. The explanation may be that people who are stressed by working in a public place and feel that their performance can be monitored or even filmed by bystanders are also more aware of their actions and therefore pay more attention to possible mistakes.

Communication problems with clinics or nursing homes seemed to correlate with higher odds of negative safety outcomes. Burghofer et al. [48] stated that communication failures account for the majority of unanticipated adverse events in patients in emergency medicine, which in our study is the case for communication with clinics and nursing homes, but not with regard to the EMS station, control center and police or fire department. Communication with clinics and nursing homes pose typical interfaces in emergency medicine where patient handoffs take place. Apker et al. [49], who investigated the communication between physicians in emergency medicine and in the emergency department during those patient handoffs, also found interfaces to be a particularly predisposed for communication errors resulting in lack of patient safety. Leonard et al. [50] found that effective teamwork in nurses and physicians supported by standardized tools and behaviors can enhance patient safety. Such standardized tools are, however, rarely implemented, especially at interfaces with clinics or nursing home, since EMS workers cooperate with various clinics and nursing homes every day. There is rarely a standardized procedure of patient handover to follow, even though clinics might have such procedures implemented among their employees [51, 52]. Those unclear procedures as well as unclear task allocation could lead to more negative safety outcomes. This is in line with rather few participants of our study reporting communication problems at the EMS-station, where it is more common to have standardized procedures that are followed by the EMS workers who are employed there.

Accordingly, frequent switching of workplaces was also significantly associated with a higher number of NSOs. Through constantly changing workplaces, EMS workers have to adapt to new or different processes and the way situations are handled in different places.

Negative safety outcomes were also associated with communication problems with patients’ relatives, but not with the patients themselves. This could be due to the fact that patients are in a critical condition and unable to communicate while their relatives are agitated while trying to act in the patient’s best interest.

Overall, we found that psychosocial working conditions showed a fairly consistent and pronounced pattern of associations with perceived impairment of patient care due to stress. This could be explained by the fact that “impairment of patient care” does not necessarily refer to actual (major) errors at work. Instead, it may also be considered to refer to poorer interaction and impatience with patients, which have been shown to be related to psychosocial working conditions in other health professions [53]. This additional dimension of patient care is not reflected by our measures pertaining to important errors or negative safety outcomes in the EMS-SI.

### Recommendations for practice

One major issue identified in this study is communication problems at interfaces. If clearer procedures were used at every interface with EMS workers, it could not only minimize stress due to miscommunication, but may also increase patient safety. An already implemented procedure is the “WHO safer surgery checklist” or “team time out” used in surgery [54]: a simple sequence of standardized information about the patient and procedure is stated and confirmed with the whole team at the beginning of the surgery. This procedure has been associated with a decrease in avoidable medical errors, patient morbidity, patient mortality, and surgical complication rates [55]. The frequent switching of teammates while working in teams of two leading to less team familiarity could be improved with team-building activities or, if possible, more consistent composition of teams.

The high prevalence of legal insecurity being perceived as a stressor in this study as well as its significant association with impairment of patient care highlights the urgent need for improvement in this area: a clearer distribution of tasks as well as a change in the law with legitimation for actions could help EMS workers stay legally protected especially in emergency situations. Since 1st of January 2023, “Notfallsanitäter*innen” in Germany are allowed to perform measures on patients until the physician is arriving, if they are appropriately trained and if those measures are necessary to prevent danger to life or substantial consequential harm to patients [56]. Although this is viewed as a step in the right direction, EMS workers still call for clearer laws and more standardized regulations throughout the different regions in Germany [57]. 

### Recommendations for research

As mentioned above, our findings suggest that the recently changed and clearer legal framework could reduce work stress in EMS workers and thereby improve patient care. It is yet to be investigated to what extent this change of law might have an impact on legal insecurity being perceived as a stressor as well as a possible improvement of patient care.

As mentioned above, association analyses yielded partly wide confidence intervals and thus our study possibly did not provide sufficient statistical power to detect associations of modest magnitude. Further research with a higher number of participants is needed to address this gap. Further research in the form of longitudinal as well as qualitative studies is needed to identify in detail where and how problems in communication occur. To determine possible opportunities for optimization, development and testing of different standardized procedures at interfaces is suggested. Finally, research is needed to further explore the correlation of work stress with other dimensions of patient care (e.g., social interaction with patients).

### Strengths and limitations

In this study, we were able to identify relevant stressors among EMS workers in Germany and thereby to describe stressors beyond existing work stress models. Through cooperation with the DBRD and the distribution of our questionnaire through their social media channels, we were able to recruit participants from different employers across Germany. The age distribution in our sample was overall comparable to estimates of the German Federal Statistics Office for EMS workers In Germany [58] with a slight overrepresentation of participants under 40 years (72.5% vs. 62.1%). Potentially, older members of the DBRD were not reached through the online distribution of the survey. Fewer female EMS workers participated than expected based on the numbers of the German Federal Statistics Office (16.5% vs. 34.1%) [58] and the known proportion of female members in the DBRD (25%).

One may speculate that DBRD members represent a specific sub population of EMS workers who respond differently as compared to the full EMS worker population in Germany. Members may be politically more engaged and more likely to expect that specific answers or results in the survey affect political action to improve working conditions for EMS workers. This expectation may have affected their reporting behavior. Furthermore, we do not know if or to what extent work stress may have played a role in participation and reporting behavior. Possibly, those exposed to higher stress levels were less likely to participate (e.g. due to competing duties and little time). Alternatively, those with high work stress levels may be overrepresented when they felt more addressed by the survey. Additional limitations of our study include that no exact response rate could be calculated due to the online distribution of the questionnaire. Our study is cross-sectional and therefore the observed associations cannot be interpreted as causal. We drew on validated instruments that had been used in the EMS workers before, but also developed some parts of the questionnaire with experts of the DBRD. The latter tool did not undergo further validation though and therefore its psychometric properties remain unknown. However, through extensive discussions of the thematic scope, comprehensibility and completeness of items, validity was assumably increased. As mentioned above, association analyses yielded partly very wide confidence intervals and thus our study did not provide sufficient statistical power to detect associations of modest magnitude.

## Conclusions

In conclusion, many stressors of the EMS work environment could be identified to be relevant in this study. The most common ones were communication problems, legal insecurities, switching of the workplace as well as switching of colleagues. Patient safety was found to be impaired to different degrees according to the various measured indicators. Accordingly, the majority of the surveyed EMS workers reported at least one negative safety outcome while concerns of having made an important error and impairment of patient care were less common. Work stress according to the DCSQ as well as job-specific work-related stressors, especially communication and legal insecurity, were significantly associated with the perceived impairment of patient care due to work stress. At the same time, psychosocial working conditions were mostly weakly and inconsistently associated with the concern to have made an important error as well as negative safety outcomes. As communication was a major stressor, it seems necessary to identify problems and to optimize working processes especially at interfaces between emergency services and other institutions. Legal insecurity could be reduced by clarifying and defining responsibilities. Communication and familiarity between team colleagues could be addressed by more consistent composition of squads.

### Electronic supplementary material

Below is the link to the electronic supplementary material.

Supplementary Material 1

## Data Availability

The datasets used and analyzed during the current study are available from the corresponding author on reasonable request.

## Abbreviations

EMS: Emergency medical service
DCS/DCSQ: Demand-Control-Support/-Questionnaire
DBRD: Deutscher Berufsverband Rettungsdienst
EMS-SI: Emergency medical service – safety inventory
IV: intravenous
IO: intraosseous
NSO: Negative safety outcome
IQR: interquartile range
CI: confidence interval
OR: Odd’s ratio
EKG: Electrocardiogram
STEMI: ST-elevation myocardial infarction
ET: endotracheal tube
