# Supplementary material for: Psychosocial working conditions, perceived patient safety and their association in emergency medical services workers in Germany – a cross-sectional study
Source: BMC Emerg Med. 2024 Apr 14;24:62. doi: 10.1186/s12873-024-00983-2 (PMC11017549; doi:10.1186/s12873-024-00983-2)
Supplement: Supplementary file 1 — Supplementary Material 1 [file 12873_2024_983_MOESM1_ESM.docx]

**Additional file 1**

**Table A:** Additional items measuring job-specific stressors of emergency medical services workers

| **Items .** | Response format | Sources |
| --- | --- | --- |
| Communication issues happen often (e.g. during the admission, decision making, organization of transports…)  ...with hospitals/nursing homes  ...with the police station  ...with the control center  ...in cooperation with the fire department or the police  ...with patients  ...with relatives | Fully disagree / disagree / agree / fully agree | (1, 17, 18) |
| My work is complicated by these communication problems | Fully disagree / disagree / agree / fully agree |  |
| I feel stressed by having to adapt to changing locations with varying specific requirements (Different and new work procedures depending on the hospital, nursing home, etc. approached). | Fully disagree / disagree / agree / fully agree | (1, 16) |
| I feel stressed by often conducting my work in public where anyone can observe me. | Fully disagree / disagree / agree / fully agree | (9, 59) |
| I mostly have shifts on the rescue vehicle with the same coworker. | Yes / no | (1, 18, 59, 60) |
| I perceive frequently changing coworkers as a stress factor. | Fully disagree / disagree / agree / fully agree |  |
| I often feel uncertain by the unclear legal situation as to which measures I may, can or must carry out. | Fully disagree / disagree / agree / fully agree | proposed by DBRD^1^ (Frank Flake) |

^1^ German Association of Emergency Medical Service (Deutscher Berfusverband Rettungsdienst e.V.)
